# Developing Bespoke High Volume Low Complexity (HVLC) Theatre Lists With a Focus on Training to Address the Impact of COVID-19: A Pilot Study

**DOI:** 10.7759/cureus.49104

**Published:** 2023-11-20

**Authors:** Sarah Zhao, Alex Rothnie, Akriti Nanda, Tarak Chouari, Sarah Ashraf, Stella Vig

**Affiliations:** 1 General Surgery, Kingston Hospital National Health Service (NHS) Foundation Trust, London, GBR; 2 General Surgery, University Hospital Lewisham, London, GBR; 3 General Surgery, Croydon University Hospital, London, GBR; 4 General Surgery, Kingston Hospital NHS Foundation Trust, London, GBR

**Keywords:** surgical education, high volume low complexity, waiting list recovery, covid-19 pandemic, surgical training

## Abstract

Introduction

The COVID-19 pandemic has had an unprecedented impact on both healthcare delivery and surgical training. There have been significant efforts to manage the growing elective waiting list backlog whilst addressing the training deficit. We outline a successful pilot high volume low complexity (HVLC) program held at the Croydon Elective Centre between 2021-2022 which aimed to amalgamate training and elective recovery.

Methods

Two pilot HVLC training lists were carried out in June 2021 and March 2022. Three parallel theatre lists on each date were supervised by a single consultant floor trainer. All lists followed a standard pre-defined HVLC protocol. Trainees and trainers were invited to participate and encouraged to utilize these lists to sign off relevant work-based assessments. HVLC cases included hernia repairs and simple lesion excisions. Patient, theatre staff, and trainee experiences were collated via questionnaires.

Results

A total of one consultant supervisor, six trainers, and eight trainees participated in the pilot with a total of 34 elective procedures performed on 29 patients. The mean patient age was 52.4 years with 8 out of 29 patients being female. Of these patients 41.4% were American Society of Anaesthesiologists (ASA) Classification one, 51.72% were ASA two and 6.9% were ASA three. No patients to date were readmitted to the hospital post-operatively or presented with post-operative complications. One hundred percent of trainees felt satisfied with the training and would recommend it to a colleague.

Conclusion

The training deficit that developed during the first COVID-19 pandemic wave has been compounded by the second and third waves, and trainees are concerned that further waves are anticipated. Returning to operating is vital and our approach has been shown to improve training, whilst maintaining patient safety and accelerating elective waiting list recovery.

## Introduction

The COVID-19 pandemic has had an unprecedented impact on surgical training and healthcare delivery in the United Kingdom (UK) and worldwide. The initial impact of the COVID-19 pandemic resulted in the mass redeployment of trainees to support critical care and medical services. Furthermore, there was almost complete cancellation of elective services due to a lack of capacity within National Health Service (NHS) resources. Whilst the NHS is recovering from the COVID-19 pandemic, there remains a training deficit where many trainees have lost experience and exposure required in both technical and non-technical skills [1-3].

The elective waiting list has also increased from 4.43 million patients prior to the pandemic to 7.68 million as of July 2023 [4]. There have been significant innovative efforts to build resilience for further waves of COVID-19 as well as winter pressures. Examples include separating urgent and elective care facilities via the surgical hub model of “hot/cold” operating sites, making effective use of independent sector capability, and the development of high-volume low complexity (HVLC) operating lists. The Surgical Royal Colleges and Training committees have emphasized the ‘No training today, no surgeons tomorrow’ ethos where every opportunity should be used to train future surgical colleagues [5]. This has resulted in an increased emphasis on balancing service provision and training and can potentially be achieved through planned training lists. These lists have a focus on specified learning outcomes agreed in advance by the team. In particular, the HVLC program as part of the Getting it Right First Time Initiative ​(GIRFT) amplifies the principle of standardization, with aims to streamline theatre processes, reduce patient turnover time, reduce length of stay, and relieve waitlist pressures [6]. 

The Croydon Elective Centre (CEC) was set up at Croydon University Hospital (London, UK) in July 2020 to facilitate safe and efficient elective operation even through the COVID-19 pandemic. This paper outlines a successful pilot HVLC programme held at the CEC across 2021 - 2022 which aimed to amalgamate training recovery with elective recovery. The objective was to train junior trainees, support senior trainees as independent practitioners, and deliver high-volume elective service. Preliminary results from this article were previously presented as a short paper at the 2022 Association of Surgeons of Great Britain and Ireland (ASGBI) Annual Congress on 3rd May 2022.

## Materials and methods

A pilot HVLC study titled ‘Hernia Fest’ was carried out across two dates on 12th June 2021 and 26th March 2022 (Saturdays) in the Croydon Elective Centre (CEC). The CEC was comprised of ten operating theatres, a pre-operative admissions unit, an adult and pediatric day case unit, an inpatient elective ward, a level 1.5 post-operative critical care unit, and a coronary catheter suite. Together these formed a dedicated ring-fenced “clean” surgical hub within the existing hospital [7]. There was a strict infection control policy in place including pre-operative COVID-19 testing and a dedicated entrance separated from the main hospital.

All cases were selected as appropriate HVLC day case procedures and where possible, similar cases were grouped within sessions to improve theatre utilization and efficiency. Types of procedures included inguinal hernia repair, umbilical hernia repair, paraumbilical hernia repair, lipoma, and simple lesion excision. Three general surgical parallel theatre lists on each date were supervised by a single Consultant floor trainer with a particular interest in surgical education. Each list consisted of two sessions and was manned by an approved senior surgical trainee (minimum one per list) and a junior trainee (minimum one per list). Senior surgical trainees were middle-grade surgical registrars who were deemed competent by the supervising consultant to act as independent or supervised trainers for the selected procedures. Junior trainees included foundation year two doctors, core surgical trainees, and trust grade "senior house officers". All participants were current or previous employees of the trust. The supervising Consultant was responsible for case selection. All theatre staff including trainees were remunerated financially for their time. Trainees and trainers were asked (not mandatory) to participate and all involved had no other concurrent commitments during the day such as on-call emergency or ward cover. Trainees were encouraged to utilize these lists to sign off mandatory critical conditions, index procedures, and other relevant work-based assessments in their training portfolios.

A standard pre-defined protocol for the list was used, outlined in Table 1 below. The pre-list brief was in addition to and not in replacement of the standard World Health Organisation (WHO) team brief. Patient safety remained a priority and provision of training during each case was at the trainer’s discretion. All patients were aware that trainees would be operating during the lists. Patient, theatre staff, and trainee experiences were collated via qualitative questionnaires which were completed before and after the theatre list.

**Table 1:** Standard pre-defined protocol for HVLC list at “Hernia Fest”

Order	Protocol step
1	Pre-list brief given to all trainers, trainees, and theatre staff to identify training needs and review caseload.
2	7.30 am pre-operative consenting and review of patients.
3	Send for the first patient at 8.15 am, with the first patient on the table for 8.30 am.
4	All patients are to be operated on the trolley without muscle relaxants.
5	Staggered lunch break or 30-minute break for lunch.
6	Standardized discharge summary and post-operative medication. A hospital contact number was provided to facilitate patient-initiated follow-up.
7	End-of-day debriefs with trainers, trainees, and theatre staff.

## Results

Across the two ‘Hernia Fest’ pilot dates, a total of six trainers [specialty trainee (ST)3 to ST8 registrar level] and eight trainees [foundation year (FY)2 to core trainee (CT2) level] participated. The minimum required trainee and trainer numbers for each date were fully met. A total of 34 procedures on 29 patients were carried out across six lists (12 sessions) with an average of 2.8 cases per session. See Table 2 for a summary of procedure types. There was one case cancellation across the two dates due to patient choice on the day of surgery (unrelated to patient knowledge of the training list). All cases were managed as training cases and similar procedures were clustered within lists. Table 2 shows a summary of patient characteristics. No patients were readmitted to the hospital post-operatively, two patients presented to the emergency department (ED) on day four and day 12 post-operatively respectively with pain at the procedure site but did not require intervention or surgical review. These were discharged directly from the emergency department. No patients have been represented with hernia recurrence to date or other complications of note.

**Table 2:** Summary of patient characteristics ASA - American Society of Anaesthesiologists

Patient characteristic	Result
Age (mean)	52.4 years
Female	27.6%
ASA Score 1	41.4%
ASA Score 2	51.72%
ASA Score 3	6.9%
Case type	
Inguinal hernia	17
Umbilical hernia	2
Paraumbilical hernia	5
Lipoma	9
Other simple lesion	1

Trainee satisfaction was 100% and all trainees would sign up for a further “Hernia Fest” list. One hundred percent of the trainees and trainers would recommend “Hernia Fest” to a colleague. About 87.5% of the trainees strongly agreed and 12.5% agreed that they felt more confident with the principles of hernia repair and that their basic surgical skills had improved as a result of the training list. Around 75% of trainers strongly agreed and 25% agreed that their trainees had improved throughout the theatre list. Senior trainees enjoyed the responsibility of running an independent list but with consultant support available as needed. By the second date, one junior trainee (CT2) was deemed already competent in low complexity lipoma cases and was therefore progressed to become a trainer for this procedure (at the discretion of the supervising consultant). One hundred percent of theatre staff enjoyed the lists and wished to participate in future lists. The consensus was that the lists did not feel pressured and that there was scope for further caseload. 

Examples of trainee/trainer feedback comments included: “How training should be”, “Creating an environment and lists focused on training makes a huge difference”, “Really fulfilling to watch the trainee improve throughout the day” and “Excellent set up for training”.

Patient feedback forms were collected as patients were discharged the same day from the elective ward. One hundred percent of patients felt confident in their treatment at the Croydon Elective Centre (CEC). One hundred percent would recommend the CEC to a relative or friend. Examples of patient feedback comments: “I was impressed with all the precautions taken prior to coming in”, “I did not have to wait long for my operation thanks to the elective center” and “The team gave me great confidence in what was to come”.

## Discussion

The key components of the HVLC training pathway are to identify training opportunities and HVLC procedures, identify trainers, planning of theatre lists and outline training objectives specific to each trainee.

Identify training opportunities and HVLC procedures

The tension existing between service provision (including National waitlist pressures) and surgical training is likely to become more evident as elective restoration continues [3]. There will be innovations as to how these HVLC parallel training lists are implemented and it is important that innovation is permitted, understood, and captured. Such recovery training lists and waiting list initiatives may occur in current NHS training hubs, new surgical hubs, or the independent sector. Surgical procedures can be divided into low, ​medium, and high-complexity cases. These procedures can also be divided into low and high-volume activities based on elective waiting lists. Categorization of these procedures allows a delivery plan for training to be developed alongside service needs. Appropriate training procedures arise from where low-complexity and high-volume categories intersect. For general surgery, defined HVLC procedures may include inguinal hernia, umbilical hernia, paraumbilical hernia, laparoscopic cholecystectomy, simple lesion, or lipoma excision [6]. Procedures should be ideally day case, low complexity, and ASA Score one or two candidates. Each surgical department should be aware of upcoming HVLC theatre lists and should be encouraged to identify appropriate training case opportunities for these lists in outpatients.

Identify trainers

Excellent training will match the right operation with the right trainee and the right trainer. The identification of excellent trainers is essential to the HVLC program and requires planning, organization, and partnership with senior managers and educators. All participants must meet professional trainer standards [5], but some trainers may be good ‘finishing’ schools for the more competent trainee while others are more adept at training the core trainee in basic surgical skills. There is also an opportunity to retain senior trainers who are peri- or post-retirement, who can add value to service and training. The new Intercollegiate Surgical Curriculum Programme (ISCP) curriculum mandates that trainees provide evidence not just in operative numbers and competence but also as independent providers of care, with assessments of Generic Professional Capabilities (GPCs) and Capabilities in Practice (CiPs) [8]. These competencies include the trainee’s ability to manage an operating list. It is important to uphold the concept of trainees as trainers, however, there must be an assurance that non-consultant trainers are competent and have evidence of completed level four procedure-based assessments (PBAs) as per the curriculum for the indexed HVLC procedure as well as adequate experience evidenced by the surgical logbook. It is strongly mandated that a senior Consultant trainer is always on hand to provide situational awareness, crew resource management, and deal with unexpected scenarios within the parallel list framework. Recognition of those who provide training should be incorporated into job plans as educational activities and can form part of the appraisal process.

Planning of theatre lists

Surgeons have a professional responsibility to train junior colleagues where possible. Clinicians should provide service and training ensuring that activity is balanced, and streamlined and training time is accounted for within the service. Planning of theatre lists is therefore vital to balance training and efficiency. Grouping similar operations together improves utilization as the theatre is set up to manage the workload [9]. This also ensures that trainees have repetitive practice in an operative procedure and can concentrate on the rapid acquisition of skills. Repetitive practice is well regarded in terms of learning new skills, consolidation of those skills, and improvement in clinical practice [10,11]. The other advantage of this pilot was that trainers were able to provide real-time concurrent live feedback during the case as well as summary feedback on completion via PBAs. The value of concurrent and summary feedback is well-established in surgical training [12-15]. Although not evaluated during this pilot, these lists can also be adapted to incorporate anesthetic trainees with parallel multi-disciplinary training opportunities. Anesthetic colleagues should be involved in planning the list and the potential for case cancellation should also be anticipated and accounted for. 

Outline training objectives specific to each trainee

The pre-list briefing is vital for the success of the parallel lists, as it enables signposting of training opportunities to all members of the theatre team and matching of trainees to appropriate cases (based on specific pathology presentation, competency, and individual training needs). Training should no longer be considered as ‘skin to skin’ in all cases but focus on individual trainee needs and different parts of each operation could be performed by different level trainees. This ensures that junior and senior trainees with different training needs can develop competencies within the same case and maximize training yield. Junior surgical trainees are a heterogeneous cohort in terms of skill set and experience, thus multiple trainees can be paired to any given training list to ensure shared high-yield learning. This again highlights the importance of matching appropriate trainees with appropriate cases to ensure high-yield training and patient safety. The key educational objectives identified for senior trainees included learning to become a trainer, an independent practitioner, and managing an operating list. Likewise, for junior trainees, the objectives included learning to become a surgeon, a patient advocate, and undertaking a low-complexity hernia repair. 

Limitations

We do recognize limitations within this study and the delivery of HVLC lists. Running these lists on a Saturday confers the advantage of fewer delays in theatre recovery flow, minimal bed issues (in the context of ring-fenced elective surgery wards), and no concurrent clinical commitments for trainees or trainers. However, an individual with weekend commitments or dependents for example may not be able to take full advantage of these opportunities. These additional lists should not be in lieu of regular training opportunities, but the concept may be adapted to a protocol that would also work during usual contracted hours. This would help improve accessibility and equal opportunity. Feedback has long been recognized for its positive impact on surgical knowledge, performance, and skills training [11,14,15]. Improvement in trainee performance throughout any given list was not objectively measured and this is an identified limitation. In future HVLC training lists, it would be both interesting and informative to carry out a linear and more objective assessment of trainee performance using validated global rating scales and other forms of operative performance analysis (e.g. hand motion analysis, operative time)[16]. In our pilot study, the trainee was encouraged to do their own reading prior to the list of relevant anatomy and technical procedure steps. However, educational yield could be further improved in the future by providing participating trainees with pre-list educational material to align theoretical knowledge with operative practice. It is acknowledged that the study includes a relatively small sample size over a short timeframe and further research is required to confirm effectiveness. However, this initial framework and its preliminary outcomes can highlight the viability of HVLC lists as training lists and can certainly be adapted to larger-scale programs.

## Conclusions

There is unprecedented need to balance service and training demand as part of the COVID-19 pandemic recovery. The ‘training deficit’ that developed during the first pandemic wave has been compounded by the second and third waves, and trainees are concerned that further waves are anticipated. Returning to operating is vital and our approach has been shown to improve training, whilst maintaining patient safety and accelerating elective recovery.
